# RNA-Seq analysis of blood meal induced gene-expression changes in *Aedes aegypti* ovaries

**DOI:** 10.1186/s12864-021-07551-z

**Published:** 2021-05-27

**Authors:** Dilip K. Nag, Constentin Dieme, Pascal Lapierre, Erica Lasek-Nesselquist, Laura D. Kramer

**Affiliations:** 1 Arbovirus Laboratory, Wadsworth Center, New York State Department of Health, Slingerlands, NY 12159 USA; 2 Bioinformatics Core, Wadsworth Center, New York State Department of Health, Center for Medical Science, Albany, NY 12208 USA; 3 Department of Biomedical Sciences, State University of New York, School of Public Health, Albany, NY 12208 USA

**Keywords:** *Aedes aegypti*, RNA-Seq, Differential gene expression, Blood meal, Egg development

## Abstract

**Background:**

Transmission of pathogens by vector mosquitoes is intrinsically linked with mosquito’s reproductive strategy because anautogenous mosquitoes require vertebrate blood to develop a batch of eggs. Each cycle of egg maturation is tightly linked with the intake of a fresh blood meal for most species. Mosquitoes that acquire pathogens during the first blood feeding can transmit the pathogens to susceptible hosts during subsequent blood feeding and also vertically to the next generation via infected eggs. Large-scale gene-expression changes occur following each blood meal in various tissues, including ovaries. Here we analyzed mosquito ovary transcriptome following a blood meal at three different time points to investigate blood-meal induced changes in gene expression in mosquito ovaries.

**Results:**

We collected ovaries from *Aedes aegypti* that received a sugar meal or a blood meal on days 3, 10 and 20 post blood meal for transcriptome analysis. Over 4000 genes responded differentially following ingestion of a blood meal on day 3, and 660 and 780 genes on days 10 and 20, respectively. Proteins encoded by differentially expressed genes (DEGs) on day 3 include odorant binding proteins (OBPs), defense-specific proteins, and cytochrome P450 detoxification enzymes. In addition, we identified 580 long non-coding RNAs that are differentially expressed at three time points. Gene ontology analysis indicated that genes involved in peptidase activity, oxidoreductase activity, extracellular space, and hydrolase activity, among others were enriched on day 3. Although most of the DEGs returned to the nonsignificant level compared to the sugar-fed mosquito ovaries following oviposition on days 10 and 20, there remained differences in the gene expression pattern in sugar-fed and blood-fed mosquitoes.

**Conclusions:**

Enrichment of OBPs following blood meal ingestion suggests that these genes may have other functions besides being part of the olfactory system. The enrichment of immune-specific genes and cytochrome P450 genes indicates that ovaries become well prepared to protect their germ line from any pathogens that may accompany the blood meal or from environmental contamination during oviposition, and to deal with the detrimental effects of toxic metabolites.

**Supplementary Information:**

The online version contains supplementary material available at 10.1186/s12864-021-07551-z.

## Background

Mosquito-borne pathogens are responsible for some of the widespread infectious diseases worldwide, such as malaria, encephalitis, filariasis, dengue fever, and yellow fever [1, 2]. Since there are no antiviral drugs or safe and effective FDA-approved vaccines against several medically important pathogen-associated ailments, vector-control strategies remain the only effective route to prevent a disease outbreak. Consequently, mosquitoes became the object of intensive investigations in order to develop novel vector-control strategies. The availability of the mosquito genome sequence provides an excellent opportunity to identify host gene targets to control pathogen transmission.

For anautogenous mosquitoes, the vector competence for transmitting a pathogen is essentially linked with their reproductive strategy, as the female normally depends on a vertebrate blood meal as a source of nutrition to produce a batch of eggs [3]. The cycle of blood feeding, egg development, and egg laying is collectively known as the gonotrophic cycle. After each gonotrophic cycle, mosquitoes return to their host-seeking stage for another blood meal. Mosquitoes that acquire pathogens during the first blood meal may transmit the pathogen to an uninfected host during these subsequent blood meals. In addition to this horizontal mode of transmission, with some viruses, the pathogens can be transmitted vertically to progeny via infected eggs [4–12]. Vertical transmission becomes important for pathogen maintenance during adverse environmental conditions, or when the number of susceptible vertebrate hosts is rare due to herd immunity or vaccination.

In *Aedes aegypti*, an anautogenous mosquito, the pre-blood meal period in the first gonotrophic cycle also includes the post-eclosion development period, which persists from 72 h to until the uptake of the first blood meal. Oogenesis in the mosquito ovary begins post-eclosion, but the oocyte growth is attenuated at a resting stage until the ingestion of a blood meal after which egg development continues until oviposition (i.e., egg-laying). In the post blood-meal (PBM) period, mosquitoes use about 20% of the blood nutrients to produce egg components within 48 h and another fraction to carry out intense biosynthetic activities, then excrete the rest [13, 14]. It takes about 72 h to complete the egg development during the PBM period. Protein-rich blood meal is required for oocyte development and vitellogenesis, during which yolk constituents (both protein and lipid) generated in the fat body are taken up by oocytes for storage and later use during embryogenesis. Vitellogenesis and oogenesis require a high level of coordination of molecular events in the fat body and ovary [3]. Multiple hormones are involved in this coordination process. Newly emerged females produce a large amount of juvenile hormone, which prime the fat body for the synthesis of vitellogenin, the precursor to the major yolk protein vitellin, and initiate limited ovarian follicle growth to its pre-vitellogenic resting stage [15]. A blood meal triggers the release of ecdysone by the ovaries; fat body cells take up ecdysone and convert it to 20-E, which triggers the activation of transcription of vitellogenin genes, coding for egg-yolk proteins, and other genes, the products of many of them are incorporated into eggs [16–18].

Clearly, a complex series of physiological events occurs in multiple tissues (e.g., midgut, fat body, and ovary) following blood meal ingestion. RNA-Seq analysis provides a useful tool to analyze changes in gene expression in the whole organism as well as in pertinent tissues [19, 20]. Comparing gene expression patterns at various time points between sugar-fed and blood fed mosquitoes and tissues, one can identify the organism’s or tissue-specific responses to the blood meal. Previous studies used RNA-Seq, microarray, and EST analyses to identify differentially expressed genes in response to blood feeding in *Anopheles gambiae, A. aegypti,* and *Aedes albopictus* mosquitoes and in tissues, such as midgut and reproductive tissues [14, 21–29]. Similar approaches have also been used to investigate the mosquito’s response to pathogen infection by several investigators [30–41]. Here, we used RNA-Seq to analyze differential gene expression following a blood meal at three time points (Days 3, 10, and 20) in *A. aegypti* ovaries without eggs. Previous transcriptome analyses in *A. aegypti* ovaries were carried out at various time points PBM until 72–96 h (i.e., the duration of the gonotrophic cycle) and also during embryonic development. In these studies, gene expression at late time points in the gonotrophic cycle was monitored in gravid ovaries. Here, we analyzed gene expression in ovaries without the eggs. Mosquitoes are expected to return to the pre-blood meal stage following each gonotrophic cycle. Our results indicated that although gene expression patterns following the gonotrophic cycle at late time points do not completely match with that of the non-blood fed (i.e., sugar fed) control mosquito ovaries, most differentially expressed genes (DEGs), however, return to the sugar-fed control level. In addition, several detoxification and defense-specific genes are also expressed at the early time point, suggesting that ovaries become prepared to avoid the ill effects of the blood meal derived toxic metabolites or to effectively deal with the pathogens that may accompany the blood meal.

## Results and discussion

### RNA-Seq analysis of *A. aegypti* ovary transcriptome

We carried out experiments to determine the ovaries’ response to blood meal ingestion by RNA-Seq analysis. Mosquitoes were fed with sheep blood and engorged mosquitoes were separated in cardboard containers. Sugar-fed mosquitoes were used as controls. Ovaries were collected from sugar-fed (SF) control and blood-fed (BF) mosquitoes on days 3, 10, and 20 PBM. In our experiments, mosquitoes were allowed to lay their eggs by providing ovitraps following blood feeding. Consequently, by day 10, most mosquitoes in the blood-fed group had laid their eggs and returned to the non-gonotrophic stage, similar to SF females. However, in several mosquitoes there were one or few unlaid eggs in the ovaries. They were manually removed before ovary collection. RNA sequencing was carried out with total RNA extracted from pooled ovaries from SF and BF mosquitoes using Illumina sequencing technology. The above three time points were selected to determine changes in gene expression patterns during and after the gonotrophic cycle. A total of 19 samples (18 samples from three biological replicates and one additional sample (day 3 BF sample) from another independent replicate, see Methods) were sequenced. Bioinformatics analysis was carried out using the CLC genomics workbench. The total number of reads per sample varied between 48,669,332 and 72,981,770 among the 19 sequenced RNA samples (Suppl. Table 1). More than 77% of the reads mapped to the host genome, with about 94% mapping to the gene regions and 6% to the intergenic regions (Suppl. Table 1).

**Fig. 1:**
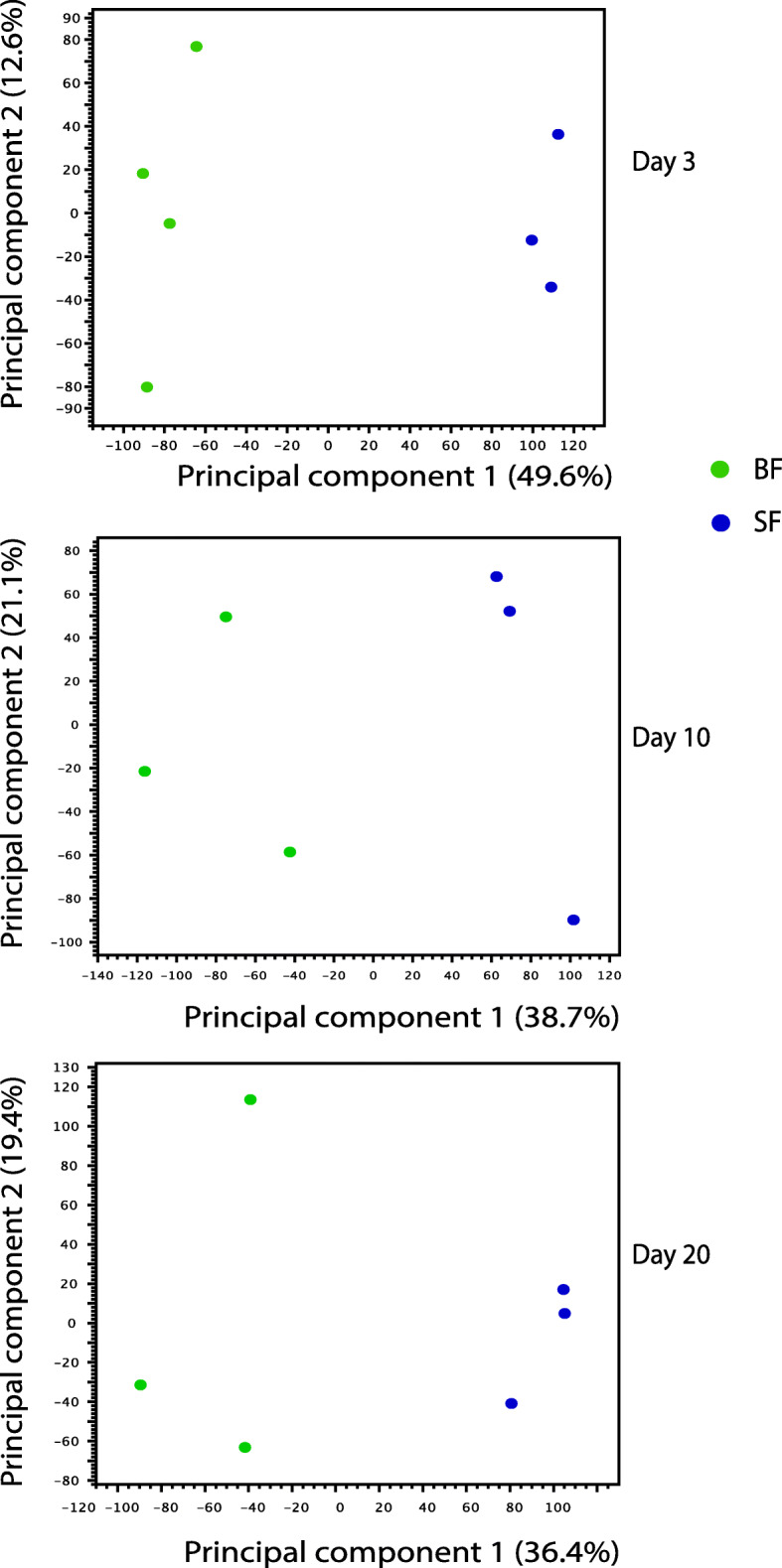
Principal component analysis of the ovary RNA-Seq data. The samples were collected at three different time points from sugar fed (SF) and blood fed (BF) mosquitoes. A total of 19 samples were analyzed by RNA-Seq (see Methods)

**Fig. 2:**
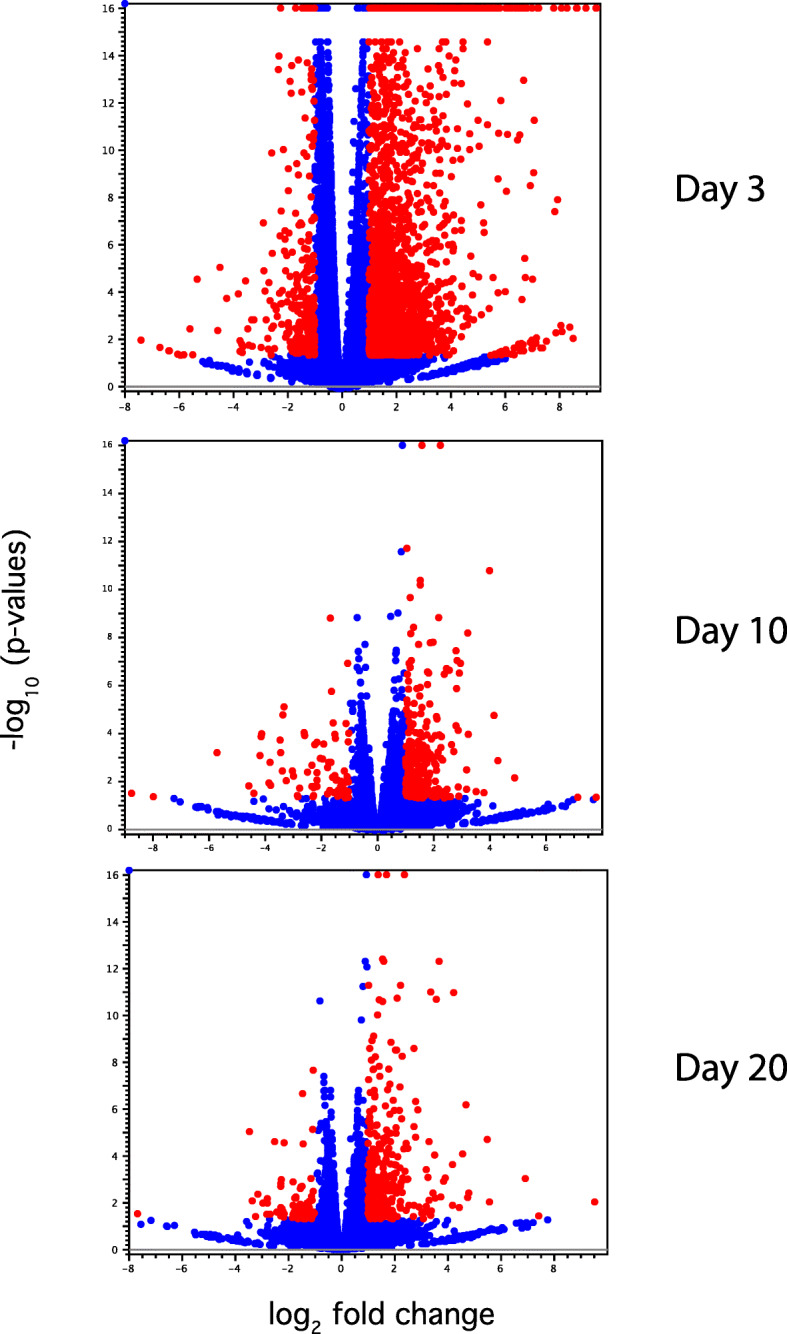
Volcano plot analysis of differentially expressed genes (DEGs) between blood fed (BF) and sugar fed (SF) ovary tissues. Red circles indicate DEGs with FDR *p*-value of < 0.05 and log_2_ fold changes > 1

**Fig. 3:**
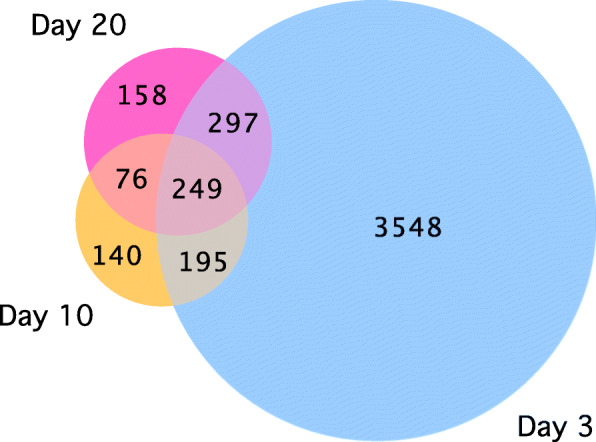
Venn diagrams showing the number of differentially expressed genes (DEGs) between blood fed (BF) and sugar fed (SF) samples at three different time points. The numbers in the overlapping areas indicate genes that were common to both or to all three different time points. The number of DEGs was highest on day 3 PBM and 249 genes were differentially expressed at all three time points

We carried out a principal component analysis (PCA) of SF and BF libraries to examine the clustering of data based on ingestion of a sugar meal or a blood meal. All biological replicates of SF and BF samples were distributed in two distinct groups (Fig. 1). Differential gene expression analysis indicated that in all time points, there were 5729 DEGs, with day 3 samples having the maximum number of DEGs (4289), and 249 DEGs were common to all three time points (Figs. 2 and 3). The numbers of DEGs on days 10 and 20 were similar (660 and 780, respectively) (Fig. 3). On day 3, there were 2743 DEGs with FDR *p*-value of < 0.05 and log_2_ fold changes > 1 (Suppl. Table 2). Under similar criteria, the number of DEGs on day 10 and 20 were 363 and 436, respectively. We have compared our RNA-Seq results with those of the previously reported transcriptome analyses of *A. aegypti* ovaries [25, 27]; the results are shown in Supplementary Table 2 and discussed below.

### Nature of DEGs in mosquito ovaries at different time points following blood meal ingestion

Since day 3 PBM had the most DEGs, we, first, focused on the nature of genes that showed differential expression patterns at this time point. Most of the DEGs are not characterized. However, we observed that several groups of genes showed altered expression patterns. One interesting group consists of odorant-binding proteins (OBPs). The term ‘odorant-binding proteins’ is used to refer to a large family of insect proteins that are exceptional in their number, abundance and diversity. The name derives from the expression of many family members in the olfactory system of insects; OBPs are involved in detection of odors and translocation of volatile chemicals to the molecular components of the olfactory receptor neuron dendritic membrane, such as odorant receptors, gustatory receptors and ionotropic receptors, which are involved in odorant recognition and transduction of volatiles into electric signals [42, 43]. Among the 13 differentially expressed OBPs, only one had a 13-fold reduction in expression over the SF control, and the rest showed overexpression ranging from 2 to 244-fold (Table 1). Many odorant receptors also had differential expression patterns (Suppl. Table 2).

**Table 1:**
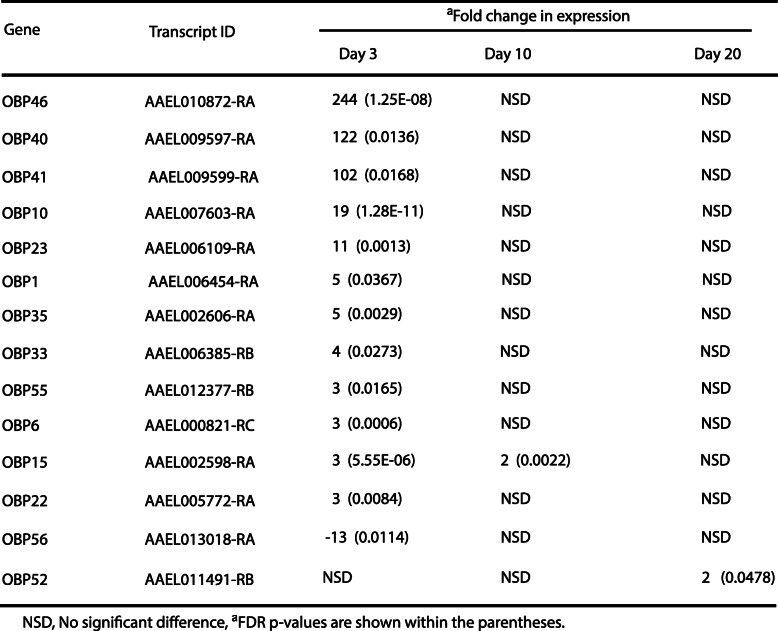
Differentially expressed odorant binding proteins (OBPs)

Previously, Akbari et al. [25] and Matthews et al. [27] studied gene-expression patterns in *A. aegypti* ovaries at various time points until 96 h PBM. Several genes that exhibited differential expression patterns in gravid ovaries were also differentially expressed in our system (Suppl. Table 2). Akbari et al. [25] also noted highly enriched OBPs PBM (Suppl. Table 2). However, highly overexpressed OBPs were not observed by Matthews et al. [27]. We expected some differences between the two studies, as mosquitoes in their system had no access to water to oviposit [27], whereas in our case a significant number of mosquitoes had laid their eggs at the time of sample collection, and eggs, if present, were removed from the ovaries before collection. Additionally, there were differences in the time (72 vs 96 h) of sample collection. It is also possible that some differences in expression patterns between the current study and previous studies are be due to geographic origin of mosquito strains [Mexico vs Africa (Liverpool strain)] used in these two studies. It has been shown that significant changes in gene expression patterns occur in *Aedes* strains depending on the place of origin, number of generations in the laboratory, and susceptibility to dengue infection [44].

During a gonotrophic cycle, after a blood meal, the host-seeking behavior is decreased and at the same time mosquitoes’ ability to find a suitable oviposition site is increased. This is when the females are behaviorally attracted to potential oviposition sites and the associated olfactory cues. Therefore, upregulation in the expression of olfactory receptors that are more attuned to oviposition attractant compounds and downregulation of receptors that are involved in recognition of compounds for host-seeking behavior in the antenna of *A. aegypti* PBM [24, 27] is not surprising. Our results showed that several odorant receptors (Or121, Or122, Or117, Or113, and Or6) were upregulated and Or30 was downregulated in ovaries PBM (Suppl. Table 2). Differential expression of OBPs was also observed in *An. gambiae* mosquitoes between 24 and 48 h PBM [21], suggesting that mosquitoes are recovering their ability to respond to odors and/or developing their ability to find good oviposition sites. Since we are studying the expression pattern in ovaries, these results suggest that ovaries may take part in the oviposition site selection or they may perform totally different functions.

Some of the induced OBPs are known to be involved in sensitive detection of oviposition attractants. For example, *Culex quinquefasciatus* OBP1 (orthologous to OBP56 in *A. aegypti*) not only binds to the mosquito oviposition pheromone, but is also involved in the reception of some oviposition attractants [45]. Our results showed that OBP56 and the ion channel *ppk301* that controls freshwater egg-laying in *A. aegypti* were differentially expressed [46], (Suppl. Table 2). OBPs are also expressed in the male reproductive tissues and transferred to the spermathecas of females [47]; OBPs thus may be involved in delivering pheromonal messages. It is also possible that OBPs are induced in response to the stress associated with oviposition or they may have a role in oocyte development. Additional studies are necessary to elucidate the roles of OPBs in ovaries PBM.

Several members of cytochrome P450 (CYP) family detoxification genes had altered expression patterns in the BF samples. Among the most and least DEGs, *CYP325N2* had nearly 14-fold overexpression and *CYP325N1* had 2-fold under-expression (Table 2). Four glutathione transferase genes exhibited 2–4 fold overexpression in the BF samples. On day 3 PBM, a large number of defense-related genes had a differential expression pattern (Table 3). *TOLL* was enriched, 20 *CLIP* genes were up and 2 were down, 12 *LRIM* were up; Defensin genes, *GNBP* genes, and Cecropin genes were over expressed following blood meal ingestion. *HOP*, *DOME*, and *IMD* expressions were not significantly different. On day 10, *TOLL5*, 3 *CLIP* and 3 *LRIMs* were upregulated. On day 20, few more defense-specific genes compare to day 10 were differentially expressed (Table 3).

**Table 2:**
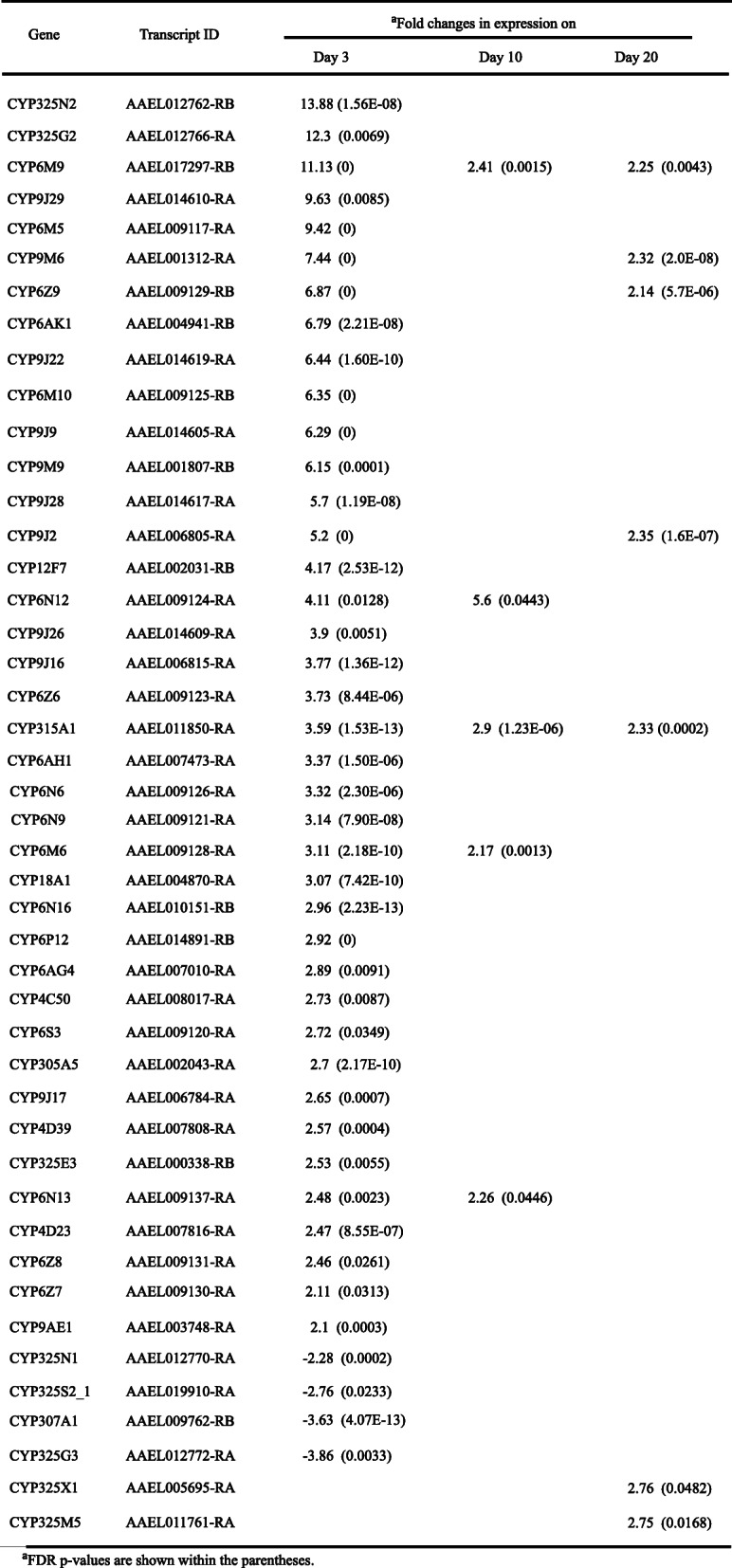
Differential expression of cytochrome P450 genes

**Table 3:**
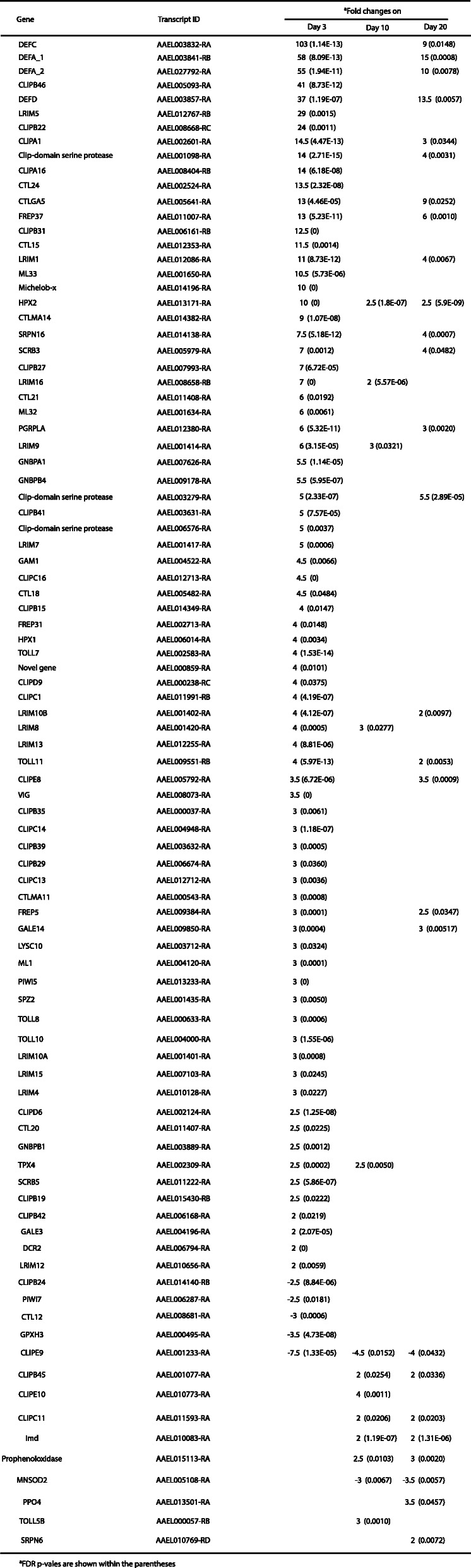
Differentially expressed defense-specific genes post blood meal

**Fig. 4:**
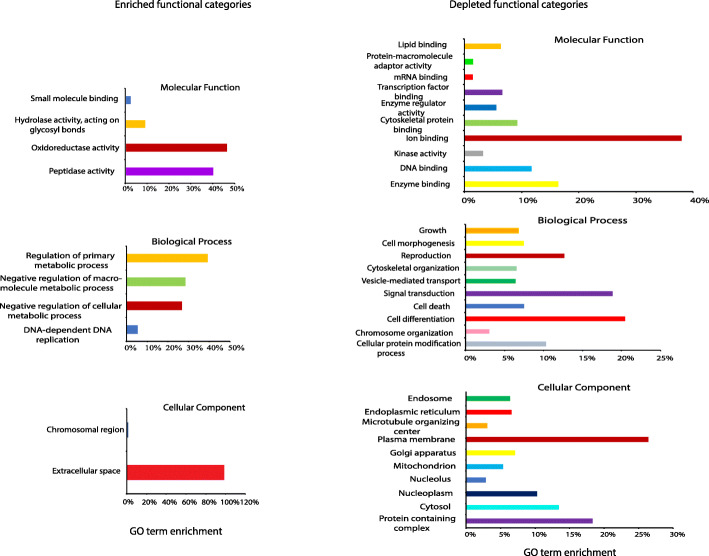
Gene ontology of differentially expressed genes (DEGs) on day 3. Enriched GO term categories are shown on the left and depleted categories are on the right. For the depleted categories only top 10 of highly significant terms are shown. For details, see Supplementary Table 3. Since GO terms for both days 10 and 20 are similar, they are not shown in the figure

The overexpression of several detoxification enzymes suggests that blood-meal ingestion not only induces gene expression for egg development, but also prepares ovaries to deal with the ill effects of any blood-associated toxins or its metabolites or to counter contamination by toxic environmental compounds during oviposition. In addition, expression of various defense-associated genes was induced following the blood meal. These results were also supported by gene ontology analysis where oxidoreductase genes were found to be highly enriched (Fig. 4 and Supplementary Table 3). Akbari et al. [25] made a similar observation in PBM ovaries. Since blood is the primary source of infectious agents, such as viruses and pathogenic bacteria, ovaries become prepared to thwart pathogens from infecting germ-line cells. In a previous study, it was observed that several immunity-related transcripts accumulated at a lower level in blood-fed mosquitoes 5 h PBM [22]. Gene expression in ovaries occurs in waves following a blood meal [21, 25]. Genes that are up- or down-regulated early in the gonotrophic cycle are not the same that occur later during egg development. It is possible that changes in expression at the whole-body level may conceal the tissue-specific changes [22], or the defense related genes are induced later in the gonotrophic cycle. It is likely that slightly overexpressed genes on day 3 could be leftover RNAs from high levels of overexpression early in the gonotrophic cycle. The expression of defense-associated genes may also result from oviposition stress or to protect the reproductive tissues from becoming infected during oviposition. Expression of immunity genes PBM is strain dependent [48, 49], which may relate to the variability of vector competence for arboviruses observed in different geographic populations of *A. aegypti*. Arbovirus infections of ovaries from infectious blood meals occur late, usually long past the gonotrophic cycle [12]. The expression of immunity genes in ovaries PBM may be one of the reasons that ovary infections occur late. It would be interesting to see the ovary’s response to an infectious blood meal.

During the PBM period, there are extreme physiological changes that require rapid coordination between tissues and between cells within the tissue. Intercellular channels, known as gap junctions, aid in the coordination of cells within tissues by the direct transfer of small molecules and ions between cells. In *A. aegypti*, six *innexin* genes (*inx1–4, 7,* and *8*) encode proteins that work as gap junctions. Similar to previous observations [50], we observed that several *inx* genes are differentially expressed (Suppl. Table 2). Among them, *inx2* was most differentially expressed with 4-fold overexpression. Three cysteine-rich venom proteins were over-enriched in day 3 samples in BF ovaries. However, their expression levels were not enriched at later time points. These venom proteins are found in animal venoms acting on ion channels [51]. One of them (AAEL000379) is also differentially expressed in *A. aegypti* following Zika virus infection [41].

On days 10 and 20 PBM, most of the genes that had an altered expression pattern on day 3 in BF samples exhibited no significantly different expression patterns compared to SF samples. For example, among the OBPs, only OBP15 had 2-fold overexpression in the day 10 sample (Table 1). Among the five differentially expressed CYP genes on day 10, four had 2-fold and one had 5-fold overexpression (Table 2). No gap junction genes had significantly altered expression patterns on days 10 and 20. Defense-related genes showed a similar trend on days 10 and 20. However, there were few more defense-specific genes differentially expressed at day 20 than at day 10. This late expression pattern of defense-associated genes could represent a response to environmental contamination or simply be due to aging.

### Gene ontology (GO)

All DEGs were subject to gene ontology analysis using Blast2GO plug-in tool of the CLC workbench. Using this analysis tool, 93, 46, and 30 gene ontology (GO) terms were identified on days 3, 10 and 20, respectively (Suppl. Table 3). These GO terms were categorized into Biological process, Molecular function, and Cellular components. The enriched GO terms included peptidase activity, oxidoreductase activity, extracellular space, and hydrolase activity acting on glycosyl bonds, among others on day 3 (Fig. 4; Suppl. Table 3). There were 83 depleted GO functional terms, including ion binding, cell differentiation, signal transduction, cell death, and plasma membrane on day 3 (Suppl. Table 3). Highly significant top 10 downregulated categories are shown in Fig. 4. On days 10 and 20, enriched GO term categories were identical: peptidase activity and extracellular region. The depleted GO term categories were also similar at these two time points (Suppl. Table 3). These results suggest that mosquitoes are ready for another blood meal.

### Differentially expressed long noncoding RNAs

**Fig. 5:**
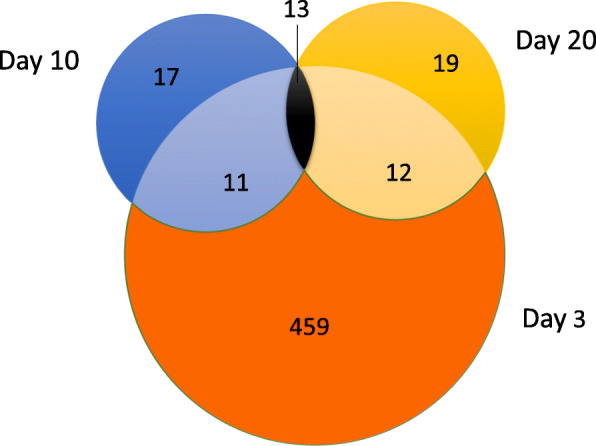
Venn diagrams showing the number of differentially expressed LncRNAs between blood fed (BF) and sugar fed (SF) samples at three different time points. Overlapping areas indicate genes that were common to both or to all three different time points. Thirteen LncRNAs were common to all three time points

In addition to the large number of differentially expressed protein-coding genes, we identified a large number of long non-coding RNAs (LncRNAs) in all three time points. LncRNAs are mRNA-like transcripts longer than 200 nucleotides but do not encode proteins. The functions of the LncRNAs, however, remain largely unknown, with a few exceptions that include LncRNAs with defined roles in embryogenesis, development, dosage compensation and sleep behavior, and they are also implicated in virus-host interactions [52–59]. The highest number of LncRNAs was observed on day 3 PBM (Suppl. Table 4). Among 2743 DEGs, 495 (18%) were LncRNAs. At 10 and 20 days PBM, the corresponding numbers were 11% (41/363) and 10% (44/436), respectively. Fold changes in the expression level ranged from 660-fold increase to an 83-fold decrease on day 3 PBM. Thirteen LncRNA were common in all three time points (Fig. 5). LncRNAs were also found to be differentially expressed in *A. aegypti* mosquitoes following ingestion of a Zika virus containing infectious blood meal [41]. It would be interesting to determine if these LncRNAs have any roles in egg development, embryogenesis, or in defense against pathogens.

### RNA-Seq validation

As mentioned above, gap-junction proteins were differentially expressed in our system as in previous studies [50]. Expression levels of genes (the ZIPs and ZnTs) encoding proteins that transport iron across membranes are increased in ovaries following a blood meal [60]. Our studies also showed that the expression of AAEL014762 (a ZIP family member) was increased nearly two-fold (FDR *p*-value 2.71 × 10^− 15^).

In addition, levels of 10 random transcripts showing differential expression PBM were tested by real-time quantitative polymerase chain reaction (RT-qPCR) (Table 4). Primers used are described in Supplementary Table 5. Transcripts were randomly chosen to include genes that showed either an enhanced or a decreased expression level. Among the genes tested, although the level of expression change does not match with that of the RNA-Seq analysis, the overall trend (over or under-expression) remained the same in both RT-qPCR and RNA-Seq analyses. Among the 10 genes tested, we included OBP46, an odorant-binding protein, to confirm that OBPs were differentially expressed following a blood meal in ovaries. OBP46 was overexpressed in both RNA-Seq and qPCR analysis. Our RT-qPCR results, thus, confirmed the RNA-Seq results (Table 4).

**Table 4:**
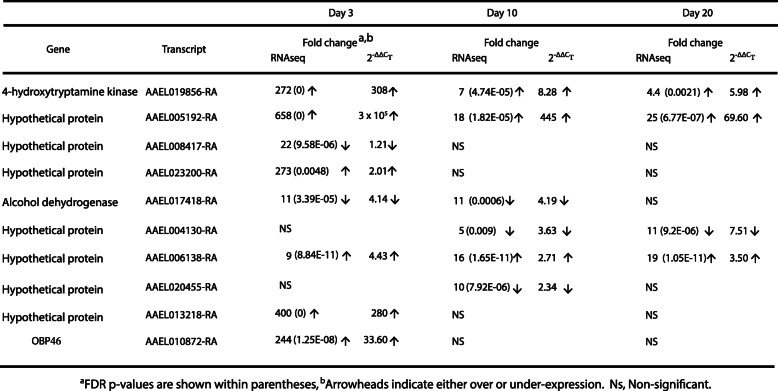
Quantitative PCR validation of the RNA-Seq ressults

Most of the DEGs on day 3 returned to the non-significant level at later time points. These results were expected since most of the mosquitoes have laid their eggs by day 10 and are ready for a second blood meal. Accordingly, gene ontology analysis confirmed that both days 10 and 20 had similar enriched and depleted gene ontology term categories. For example, both peptidase activity and extracellular region categories were enriched on both days 10 and 20. The result of our gene-ontology analysis is also similar to that of the gene expression studies by EST analysis in autogenous *Georgecraigius atropalpus* mosquito ovaries during the autogenous phase of egg production [23]. Similarities include identification of transcripts associated with intermediary metabolism and extracellular space (Suppl. Table 3).

Although most DEGs returned to the non-significant level at later time points, the gene expression pattern in blood-fed mosquito ovaries differed slightly from SF ovaries at the same time point. More than 300 genes exhibited differential expression patterns on days 10 and 20 PBM. The data presented here represent ovary transcriptomes in mosquitoes that do not take a second blood meal during a gonotrophic cycle. In nature, *A. aegypti* generally takes multiple blood meals during a single gonotrophic cycle. Each meal will trigger gene expression for egg maturation. Consequently, the pattern of gene expression observed in our studies may not represent what happens in nature. Further studies with additional blood feeding in the same gonotrophic cycle are necessary to address this issue.

## Conclusions

A large number of genes were differentially expressed following ingestion of a blood meal in mosquito ovaries when compared to SF ovaries. Differential expression is highest on day 3 PBM and most of the DEGs return to the non-significant level by day 10, although expression was not identical to the SF samples. Major categories of DEGs on day 3 included OBPs, cytochrome P-450 mediated detoxification genes, defense-specific genes among others. Gene ontology terms including, peptidase activity, oxidoreductase activity, negative regulation of cellular metabolic and macromolecule metabolic processes were significantly enriched on day 3 PBM. Since blood is the primary source of infection by viral or other infectious agents, ovaries must become prepared to deal with blood-borne or environmental pathogens to protect their germ-line cells. Studies are underway to reveal ovary’s response to infectious blood meals.

## Methods

### Mosquito feeding and rearing

All mosquito rearing was done at 28 °C. 4–7 days old *A. aegypti* (from Poza Rica, Mexico, kindly provided by Greg Ebel, CSU) were fed with warmed sheep blood (Colorado Serum Company). No additional blood meals were given for the entire study period. After feeding for an hour at room temperature, engorged females were separated in cardboard containers. A 65-mm petri dish containing water-soaked cotton covered with a wet coffee filter paper was placed inside the jar for laying eggs. All mosquitoes, both control and blood fed, were maintained with 10% sucrose and reared identically.

### Library preparation

Ovary samples were collected on days 3, 10, and 20 PBM. Ovary dissection was carried out in sterile 1X phosphate buffered saline and stored immediately at -80 °C until RNA isolation. Ovaries were collected from about 25 mosquitoes for each time point. Eggs, if present, were removed from the ovaries before collection. Total RNA was isolated using the mirVana miRNA isolation kit (Invitrogen) then treated with Turbo DNase followed by inactivation of the DNase using the supplied DNase inactivation reagent following the manufacturer’s suggestions. rRNA was depleted using the Illumina Ribo-Zero gold kit. Prior to library preparation, mosquito total RNA was quality checked using an Agilent Fragment Analyzer and Qubit Fluorescence (Invitrogen). RNA libraries were prepared following the Illumina Stranded total RNA library prep kit with Ribo-Zero Gold protocol. Following library concentration and quality control, final libraries were pooled to 10 nM and the concentration of the pool was determined using the Kapa Biosystems Universal qPCR kit. After dilution and denaturing, the pooled library was loaded onto a NovaSeq6000 S1 flow cell (PE200) for sequencing. Library preparation and sequencing were done with three biological replicates at the University of Buffalo Genomics and Bioinformatics Core. A total of 19 samples were sequenced. Eighteen samples from three biological replicates (Replicates 1, 2, and 4). There was room for an additional sample; we included a day 3 blood-fed sample from another replicate (Replicate 3). The raw sequences were subjected to quality check by FastQC.

### RNA-Seq data analysis

Qiagen CLC genomics workbench v.20 was used for bioinformatics analysis in this study. Compressed FASTQ files were extracted, trimmed, and filtered using the default parameters of the modified Mott trimming algorithm as implemented in CLC. Differential gene expression analyses were performed using the proprietary RNAseq analyses module as implemented in Qiagen CLC genomics workbench using the default parameters. The CLC RNAseq workflow uses multi-factorial statistics based on a negative binomial Generalized Linear Model (GLM) to determine statistically supported differentially expressed genes between treatments. More specifically, reads were mapped to the VectorBase reference genome (*Aedes aegypti* L 5.0) with mismatch, insertion, and deletion costs parameters set at 2, 3, and 3, respectively, with a length fraction of 0.8 in mapping parameter, where 80% of the nucleotides in a read must be aligned to the reference genome. Principal component analysis (PCA) was used for quality control, tissue contamination, problems with experimental design, and to visualize variation between expression analysis samples. Venn diagram and volcano plot were generated on the CLC workbench.

### Gene ontology

Functional annotation and gene ontology analysis were carried out using the Blast2go Plug-in tool of the CLC workbench. We used blast (SwissProt) and InterProscan to reveal the gene ontology term for the *A. aegypti* transcripts obtained from VectorBase (Aedes-aegypti- LVP_AGWG_TRANSCRIPTS_AaegL5.2.fa.gz). Fisher’s exact test was used to categorize the DEGs into Biological process, Molecular function, and Cellular components.

### RT-qPCR analysis of mRNA

To validate the RNA-Seq results, we used RT-qPCR to measure the relative abundance of 10 transcripts in ovary total RNA preparations using RNA samples from two biological replicates used in the RNA-Seq analysis and two new biological replicates. So, RNA samples from four replicates were used for RT-qPCR validation. Total RNA was prepared and treated with Turbo DNase as described above. Total RNA was reverse transcribed (200 ng in a 20 μl total volume) using superscript IV first strand synthesis system (Thermofisher Scientific). A mixture of Oligo d(T)_20_ and random hexamers was used as primers. Real time Quantitative PCR was performed using 1X powerup SYBR green master mix and 4 pM of each primer and 0.2 μl of the cDNA mixture in a 10-μl total volume. Relative abundance was determined using the Livak (△△C_T_) method. All polymerase reactions were performed in quadruplicate. The ribosomal S7 protein-coding gene was used as an endogenous reference. Primer sequences are shown in supplementary Table 5.

## Supplementary Information

**Additional file 1: Table S1.** RNA read summary of BF and SF libraries.

**Additional file 2: Table S2.** Significant DEGs between BF and SF samples at three different time points.

**Additional file 3: Table S3.** Functional categories of  DEGs.

**Additional file 4: Table S4.** Differential expression of LncRNAs at three time points.

**Additional file 5: Table S5.** Primers used for qPCR assay.

## Data Availability

All raw and processed data are deposited at NCBI Gene Expression Omnibus (GEO) with the accession number GEO GSE152209 (https://www.ncbi.nlm.nih.gov/gds/?term=GEO%20GSE152209). Gene set annotation, expression data and identified long non-coding RNAs are included as supplementary files with the manuscript.

## Abbreviations

BF: Blood fed
CYP: Cytochrome P450
DEG: Differentially expressed gene
EST: Expressed sequence tag
FDR: False discovery rate
LncRNA: Long non-coding RNA
OBP: Odorant binding protein
PBM: Post blood meal
PCA: Principal component analysis
RT-qPCR: Reverse-transcriptase quantitative polymerase chain reaction
RNA-Seq: RNA sequencing
SF: Sugar fed
