# Supplementary material for: RNA-Seq analysis of blood meal induced gene-expression changes in Aedes aegypti ovaries
Source: BMC Genomics. 2021 May 27;22:396. doi: 10.1186/s12864-021-07551-z (PMC8161926; doi:10.1186/s12864-021-07551-z)
Supplement: Supplementary file 5 — Additional file 5: Table S5. Primers used for qPCR assay. [file 12864_2021_7551_MOESM5_ESM.pdf]

Table S5. Primers used for qPCR assay

Gene primer name	Primer sequence
AAEL019856 For	GTG ATC TAC CGT GCC CAA AT
AAEL019856 Rev	GAC ATC GTA CTC GCC TAG TTT C
AAEL005192 For	CTG TGG ACT CAG TTC ATT AGG G
AAEL005192 Rev	CCT GTC TAA AGC ATC GAC TAC AA
AAEL008417 For	GAA ACG GAA GTC GCT CAA AC
AAEL008417 Rev	GCA GCC TTC ATT CGT TTC TC
AAEL023200 For	CAG TAA TCG AAG GGT GGA TTG T
AAEL023200 Rev	CGG CTC CAG CAA GTA GAT TT
AAEL013218 For	CAG GTA GCC GAA GTG TTC TAT G
AAEL013218 Rev	GCC GAC TTT GTG TAG GAT GT
AAEL004130 For	ATA CTC TGG CCT TCG CAA TC
AAEL004130 Rev	GCT GGA GAA CTC ACG TTC AA
AAEL020455 For	CAC CAG CAG ATC CAT CGT AAT
AAEL020455 Rev	GCT CCT TCT TCT TCG GTT TCT
AAEL017418 For	GGC ACA GCA GTT CCT TAG T
AAEL017418 Rev	GGT ATA GAT GGG CGT CGA AAG
AAEL006138 For	GCC ATA CTT CAC CGG CTA TAA G
AAEL006138 Rev	TTC GGC GAT TTC CTC GAT AAG
OBP46 For	CAC TCA GAA CTA CGC TGG ATA C
OBP46 Rev	GCT CTG GTG CAC TTG TCA TA
S7 For	ACC GCC GTC TAC GAT GCC A
S7 Rev	ATG GTG GTC TGC TGG TTC TT
